# Handwriting Legibility and Its Relationship to Spelling Ability and Age: Evidence From Monolingual and Bilingual Children

**DOI:** 10.3389/fpsyg.2020.01097

**Published:** 2020-06-04

**Authors:** Markéta Caravolas, Cameron Downing, Catrin Leah Hadden, Caspar Wynne

**Affiliations:** School of Psychology, Bangor University, Bangor, United Kingdom

**Keywords:** handwriting legibility, spelling, writing experience, bilingual, monolingual, predictors, orthography-specific, language-general

## Abstract

Studies of the relationship between spelling and handwriting concur that spelling skills influence the dynamic processes of handwriting. However, it remains unclear whether variations in spelling ability are related to variations in the legibility of handwriting, how important spelling skills are relative to the amount of handwriting experience afforded by an individual’s age and number of years of schooling, or to what extent this relationship may be task- and orthography-specific. We investigated these questions in a study comparing spelling and handwriting legibility in a group of *N* = 127 Welsh-English bilingual children matched in age and number of years of schooling to a group of *N* = 127 English-monolingual children, as well as to a group of *N* = 127 younger, English monolingual children matched to the bilingual group in spelling ability. All groups completed the Spelling and Handwriting Legibility Test (SaHLT) and a broader battery of literacy measures. The bilingual children were found to have poorer handwriting legibility than same age peers, and in some cases, than their younger, spelling-ability peers, suggesting that spelling ability, more so than amount of handwriting experience and years of schooling impacts handwriting legibility. This was corroborated in a series of multi-group path models, where all children’s handwriting was predicted by spelling ability more strongly than by age, and, the effect of spelling generalized across two different spelling tasks in all groups. Finally, bilingual children seemed to draw on general (Welsh) as well as on orthography-specific (English) knowledge when handwriting in English.

## Introduction

Spelling and handwriting skills, also called transcription skills (Juel et al., 1986; Berninger and Swanson, 1994), form a crucial, but to date understudied skill set in children’s writing acquisition. They are temporally closely related processes of writing production, both occurring virtually simultaneously. However, models of writing production see them as separate, dissociable skills, under the control of different systems (e.g., van Galen, 1991). Spelling is a language-based skill under cognitive control, while handwriting is generally seen as a psychomotor skill under motor control. More recent elaborations of van Galen’s model (e.g., Roux et al., 2013; Olive, 2014), the theoretical framework adopted for the present study, view the component processes of writing as a cascading and partially overlapping series of events, with spelling preceding, but also being modulated by handwriting processes. Despite their separate origins, the two skills are correlated (e.g., Berninger et al., 1992; Sumner et al., 2014), although the nature of the relationship between them is complex, and only beginning to be understood (e.g., Sumner et al., 2012; Kandel and Perret, 2015).

Handwriting comprises two aspects: the ability to write easily at speed, without undue effort and hesitation (fluency) and the ability to write clearly (legibility). Handwriting fluency is relatively easy to measure objectively as the number of units of writing produced per unit of time, for example, by the number of alphabet letters produced in 1 min (e.g., Jones and Christensen, 1999; Pontart et al., 2013; Alamargot and Morin, 2015). Other, more subtle, time-dependent behaviors that occur during handwriting and reveal the ‘real-time handwriting dynamics’ can be measured objectively thanks to technologies allowing detailed, real time tracking of pen movements (see below). Given the relative ease and measurement objectivity enabled by these tools, fluency in handwriting has been more extensively studied (Lambert and Quémart, 2019). It has been found to uniquely predict, across the developmental spectrum, the quantity and quality of text composition (Graham et al., 1997; Connelly et al., 2006; Puranik and Al Otaiba, 2012). This effect has been interpreted to indicate that when writing becomes automatic (as indexed by measures of fluency), cognitive resources are freed up for other, higher, skills of writing (Berninger and Amtmann, 2003; Berninger and Winn, 2006).

Research on handwriting dynamics has significantly moved the field forward in demonstrating how the attributes of words (such as phonology-to-orthography consistency (Roux et al., 2013; Kandel and Perret, 2015), and frequency (e.g., Delattre et al., 2006; Afonso et al., 2018) can impact processes such as the latencies (pauses prior to the onset of writing) and/or handwriting durations (time pen spent on the surface whilst creating strokes/letters/words), and pausing within words during spelling. Furthermore, studies conducted with children in various orthographies including French (e.g., Kandel and Perret, 2015), Spanish (e.g., Afonso et al., 2018), and Norwegian (e.g., Søvik et al., 1996) have focused on the influence of spelling ability on handwriting dynamics. However, these studies have used different task types (dictation vs. copying), measures of handwriting dynamics (onset latency between stimuli presentation and beginning writing, stroke duration, letter duration), and age groups, making it difficult to assess the extent and nature of the moderating effects that orthographies may have on spelling and handwriting. Studies that include direct cross-linguistic comparisons and studies of bilingualism will advance the understanding of the generalities and specifics of the spelling-handwriting relationship. The second aspect of handwriting, legibility, has been studied less extensively, in part because it is a skill more difficult to measure. Yet, the practical and educational consequences of poorly legible handwriting are arguably more pervasive than those of slow or dysfluent handwriting. Common features of poor legibility include distortions and inconsistencies in letter shape and size, poor spatial organization and spacing of letters and of words (e.g., Rosenblum, 2008). At school, children with poor handwriting are more likely to receive lower grades than those with better handwriting for comparable content, they are at greater risk of falling behind academically, and more likely to experience lower self-esteem and greater loneliness than peers with good handwriting (e.g., Feder and Majnemer, 2007). Thus, a better understanding of the development of handwriting legibility, its cognitive and motor underpinnings, as well as its relationship to spelling ability are warranted.

The complex relationship between handwriting and spelling has been the subject of a growing body of scientific investigation. In studies of skill development, a common approach is to examine how individual and group differences in spelling skills influence handwriting (e.g., Abbott and Berninger, 1993; Sumner et al., 2012). Studies comparing typically developing groups and groups with known deficits in spelling, namely children with dyslexia for whom spelling difficulties are a hallmark feature, have generally focused on the fluency and real-time dynamics of handwriting processes (Sumner et al., 2012, 2014; Kandel et al., 2017; Afonso et al., 2019; Arfé et al., 2019), but not legibility. These studies concur that, although handwriting difficulties (e.g., dysfluency due to pausing and pen movement durations) are not a core cause of dyslexia, they are concomitant reflections of dyslexic children’s weaknesses in orthographic knowledge and processing during spelling. In contrast, studies reporting a link between spelling and handwriting legibility have been mainly based on anecdotal evidence (e.g., Cooke, 2002). Martlew (1992) carried out one of the few empirical studies that considered both handwriting fluency *and* legibility in their relationship to spelling ability, in comparisons of children with dyslexia to age- and younger ability-matched control groups. An interesting finding of this study was that, while the speed-related motor dynamics of handwriting seemed more dependent on amount of writing experience, such that the younger spelling-ability-matched children wrote generally more slowly than their older dyslexic and non-dyslexic counterparts, handwriting legibility seemed more closely associated to spelling ability. That is, judges could reliably categorize older typical writers in terms of their handwriting legibility, but seemed to be at chance in distinguishing between dyslexic and ability-matched children on the basis of legibility. Martlew’s results must be considered as tentative, however, because the study included small sample sizes, and the relationships between spelling ability and speed versus spelling ability and legibility of handwriting were not compared directly.

The above experimental studies have compared groups with dyslexia to their typically developing chronological age mates. Such comparisons reveal, in theory, the gap between impaired performance caused by dyslexia and expected attainments in the absence of the disorder, given similar age and schooling experience of the two groups of participants. However, individuals with dyslexia tend to read and write less than their typically developing peers (Stanovich, 1986; Juel, 1988), and consequently many have relatively less experience and practice with both spelling and handwriting; this lesser experience may compound the expression of either or both of their difficulties. This issue is partially addressed by the inclusion of typically developing comparison groups who are younger but matched for spelling ability with the dyslexic group (e.g., Sumner et al., 2012, 2014). Such younger control groups can shed light on the extent to which spelling and handwriting skills are interlinked for those without a spelling disorder but have had (as of yet) lesser experience and practice in the two skills. However, because it is difficult to quantify how much less reading and writing experience individuals with dyslexia have had, matching for spelling ability does not entail matching for the amount of writing experience of the two groups, nor does it account for probable effects of general maturation between the younger typical and older dyslexic groups.

The role of handwriting practice and experience, over and above spelling skill, may play a particularly important role in the development of handwriting, this being a skill primarily in the motor domain. Indeed, one defining characteristic of developmental co-ordination disorder (DCD), a disorder of motor functions, is persisting handwriting difficulty (Miller et al., 2001). While studies comparing the handwriting skills of typical groups and groups with DCD are informative about the proximal impacts of motor skills on handwriting development, they also involve a comparison with a group with a neurocognitive disorder. As in studies of groups with dyslexia, comparisons with such “disorder groups” do not allow for the role of practice and experience in either spelling or handwriting to be decoupled from the effects of the disorders themselves. However, the same design can be effectively used in research with bilingual (or second language – L2) learners of a language, and it may better separate overall handwriting experience (i.e., in the main language of instruction) from spelling ability. This is because the monolingual age-matched group and the typically developing bilingual group should have had comparable amounts of writing experience in their main language of instruction, but of course not in the amount of experience they have had in writing the bilingual group’s L2. In contrast, while the bilingual group will have had more writing experience than the younger, monolingual spelling ability-matched control group, their spelling knowledge of the language in question will be comparable.

In the present study, we adopt such an approach to the investigation of the spelling-handwriting relationship, which is novel in two respects. First, we consider group and individual differences among children who are typically developing, and yet have lower spelling skills due to their bilingual, Welsh-English, education context. Specifically, we studied mid-primary-school-aged children in Welsh-medium schools where all instruction is provided in the Welsh language from 5 to 7 years of age (the North American equivalent of kindergarten to second grade) and only as of the third grade (age seven onward), English instruction is introduced. An interesting characteristic of this population is that most are bilingual speakers (but not readers) of English already in the early school years preceding formal English tuition. We compared the English spelling and handwriting skills of this bilingual group to age-matched peers, and to younger, spelling-ability-matched peers who were English monolinguals attending English-medium schools in the same region and under the same education authority. Thus, all groups were typically developing, and the age-matched (bilingual and monolingual) groups had the same amount of schooling under similar educational curricula and literacy skills targets across the primary years (Welsh Government, 2007). Their younger peers shared English monolingualism with one group, and English spelling level with the other.

It is useful to point out a few features of the Welsh orthography that have direct relevance to this study. The Welsh language uses an alphabetic orthography with almost full overlap of letters with the English alphabet (although Welsh additionally uses several diacritics), and thus Welsh-English learners’ main challenge in acquiring written English after having acquired the rudiments of written Welsh involves learning the new orthographic patterns of English. The acquisition of written English, which most children already speak, does not entail learning a new script or a new handwriting style. There are considerable orthographic differences between the languages in terms of the typical and permissible spelling patterns (graphemes), and Welsh has a more grapho-phonemically consistent orthography than English. Thus, in terms of spelling, the bilingually educated children in the present study were learning the more complex spelling system of English after several years of learning the less complex system of Welsh. Note that, in the present study, the factor of orthographic consistency was not explored *per se*.

The second novel feature of the present study is our focus on handwriting legibility, as opposed to fluency and real-time handwriting dynamics. This was done to enrich current understanding of children’s handwriting development by investigating whether, as is the case for the link between spelling and handwriting fluency, there is also a link between spelling skill and handwriting legibility, and whether this is true for learners with monolingual versus bilingual language backgrounds. Measuring handwriting legibility presents various challenges. Assessments of legibility are often globally scored, with a ‘grade’ awarded for whole texts (Rosenblum et al., 2003). These and even more fine-grained scales often suffer from relatively low reliability and validity, and they are difficult to replicate because there is an inherent element of subjective judgment in their evaluation. In an attempt to overcome these limitations, we developed a scale, the Spelling and Handwriting Legibility Test (SaHLT), which measures spelling and handwriting based on the same task. The handwriting component assessed four separable dimensions, recognized also by other scholars (e.g., Rosenblum et al., 2003) to contribute to legibility: letter formation, letter spacing, word spacing, and line alignment. We explain the design and psychometric properties of the test in the Methods section. Here, we highlight the main constructs assumed to be measured by each of the four dimensions. All four are related to motor skills, however, letter formation and word spacing are also likely related to spelling skills. Letter formation has been reported to be related to letter knowledge (Longcamp et al., 2008) an important spelling-related skill (Caravolas et al., 2012). Accordingly, we recently found strong correlations between letter formation, as measured by the SaHLT, and spelling ability; moreover, children with dyslexia were found to have poorer letter formation than typically developing children (Downing and Caravolas, 2018). We also found that low scores on the word spacing dimension were related, not only to visual-spatial distortions in spacing between words, but also to incorrect use of word boundaries (for example, spelling “around” as two words “a round”), suggesting that performance on this dimension may also be related to morpho-phonological knowledge in spelling. While letter formation and possibly word spacing are related to spelling, letter spacing and line alignment are assumed to be more reflective of motor skills.

Here, we attempted to disentangle the effects of handwriting experience, which we assume is reflected across groups by age and amount of schooling experience, from spelling ability, on handwriting legibility. We did so by examining typically developing, bilingual spellers in Welsh-English education in comparison to age- and spelling-ability-matched English monolingual counterparts. Thus, pupils learning in the bilingual context had comparable experience of handwriting to their age peers in monolingual English education, but were likely to have weaker English spelling skills in the mid-primary years (grades 3–6) due to the later start of formal tuition in this orthography. The bilingual group was anticipated to have comparable spelling skills to monolingual English children with approximately 1 to 2 years less schooling.

We hypothesized that if the association between spelling ability and handwriting legibility is mainly contingent on the impacts of effortful and error-prone spelling processes during handwriting production, then the bilingual and spelling ability matched monolingual groups should show concomitant handwriting weaknesses relative to older and better spellers. This pattern of results might be most strongly evident on outcomes for the letter formation dimension. If, on the other hand, handwriting legibility develops as a function of general, language-independent spelling and writing experience and practice, then the bilingual pupils should produce handwriting that is as legible as that of their monolingual age- and schooling-matched peers. The latter pattern may be more clearly evident on the handwriting dimensions of letter spacing and line alignment, thought to be more strongly indicative of handwriting components under motoric, and visuo-spatial control, and thereby be more amenable to general handwriting experience and practice, be it in Welsh or in English. To test these hypotheses, we carried out between-group analyzes on English spelling and handwriting legibility scores obtained from the SaHLT measure.

Furthermore, we were interested in examining whether previously reported associations between spelling and handwriting fluency and its dynamic processes (e.g., Kandel and Perret, 2015) also held for spelling and handwriting legibility. Moreover, to test the generality of this relationship, we also examined whether any relationship between spelling and handwriting legibility was only present when measured by the same task or whether it would hold across different measures of spelling. We also probed whether associations between spelling and handwriting among bilingual writers depended on the orthography in which they were writing (orthography-specific) or whether they reflected general spelling skill (i.e., Welsh and English). We addressed these questions in a series of multi-group path analyses, in which spelling ability, age (our proxy also for amount of writing experience), non-verbal ability and reading skills were included as potential predictors of handwriting legibility.

## Materials and Methods

### Participants

The participants were selected from two larger studies of typically developing children, one of *N* = 294 pupils in primarily Welsh-medium education, and one of *N* = 936 pupils in primarily English-medium education. The two studies had partially overlapping aims and hence included a number of the same assessments. All participants came from North Wales and were schooled according to similar curricula set by the Department for Education and Skills (Wales); the main difference between the cohorts was their language profile. In Wales, children attending Welsh-medium schools are taught through the medium of Welsh throughout the Foundation Phase (3 to 7 years of age). During Key Stage 2 (7 to 11 years of age), English is introduced formally, however, children still experience up to 70% of their learning through the medium of Welsh. For the present study, 127 Welsh-English bilingual (BIL) children (69 girls) in grades 3, 4, and 5 (7.8 – 10.8 years of age) were selected from three schools of the larger Welsh-medium cohort. In addition, 254 English monolingual children were selected from across six schools in the larger English-medium cohort, of whom 127 (69 girls) were in grades 3, 4, and 5 (7.8 – 10.7 years of age) and were matched to the individuals in the bilingual group according to chronological age (± 3 months) and non-verbal reasoning ability (as close to ± 3 points as was possible); this was the chronological age (CA) control group. The remaining 127 participants (68 girls) of the 254 English monolingual children, were in grades 2, 3, and 4 (6.3 – 10.1 years of age), and were matched to the bilingual children according to spelling (binary sentence spelling) ability and non-verbal reasoning (matrix reasoning) skills; this was the spelling-ability-matched (SA) control group. Details of each group’s sample size and age by grade level are provided in Table 1.

**TABLE 1:** Sample sizes, means, and standard deviations of ages in months for the bilingual, chronological-age-matched and the spelling-ability-matched groups as a function of Grade.

	**Grade**			
	**2**	**3**	**4**	**5**
**Chronological Age**				
*n*		35	46	46
Age (months)		100.60	110.61	123.61
*SD*		3.52	3.51	3.59
**Bilingual**				
*n*		35	46	46
Age (months)		101.74	110.74	123.72
*SD*		4.07	3.57	3.90
**Spelling Age**				
*n*	28	62	37	
Age (months)	80.54	97.48	110.68	
*SD*	3.18	3.66	4.06	

Regarding language profiles, all participants were asked about their language preferences in three set questions, and confirmations about their responses were sought from teachers. These self-reports revealed that, in the bilingual group, 55% of participants identified as preferentially Welsh speakers, 46% as preferentially English speakers, and 0.8% as preferring an ‘other’ language. The subgroups did not differ in terms of their English or Welsh literacy skills, however, and therefore they were considered a single language-profile bilingual group for the purposes of the present study. The participants in English-medium schools were primarily English monolingual. In response to the language preference questions, 98% of the sample identified as preferentially English speakers, and 1.6% as preferentially speakers of an ‘other’ language.

All participants completed measures of non-verbal reasoning, reading, spelling, and handwriting legibility skills in English. In addition, the bilingually educated children completed spelling and reading tests in Welsh (see Materials section). None of the participants had a statement of special educational needs. Head teachers were invited to opt into the project, while the opt-out method of consent was used in seeking parental approval because the testing was carried out in classrooms as part of the children’s main instruction. The study was conducted in accordance with the British Psychological Society Code of Ethics and Conduct.

### Materials

Descriptive statistics of the raw and standardized scores (where appropriate) as a function of group for the measures listed below are reported in Table 2.

**TABLE 2:** Descriptive statistics and group comparisons on age and literacy background measures for bilingual, chronological age-, and spelling ability-matched control groups.

**Measure**	**CA**		**BIL**		**SA**		**Group comparisons**	
	***M***	***SD***	***M***	***SD***	***M***	***SD***	***F***	**$\eta_{\rho }^{2}$**
Matching								
Age (months)	112.56	9.90	112.76	9.90	97.59	11.33	88.95***	0.32
Matrices^a^	94.99	13.94	98.93	15.33	102.52	13.41	8.16***	0.04
Sentence Spelling								
Binary	34.83	8.36	29.57	11.12	29.69	11.05	10.87***	0.05
Letter Distance	0.82	0.62	1.02	0.72	1.25	1.01	9.59***	0.05
Additional literacy								
Picture-Word Matching								
English								
Raw	37.43	9.46	44.44	11.18	31.59	10.05	55.50***	0.24
Standardized	102.30	14.29	101.46	14.06	103.79	12.74	0.81	< 0.01
Language of education^a^	102.30	14.29	101.20	15.43	103.79	12.74	0.92	0.01
Word Spelling (language of education)^a^	101.59	12.54	100.65	14.04	103.96	13.12	2.19	0.01

^a^Standard score (M = 100, SD = 15); ***p < 0.001.

#### Test of Non-verbal Reasoning Ability

We adapted the Matrices subtest of the Wide Range Intelligence Test (WRIT; Glutting et al., 2000) for group administration. This measure was included as a proxy for non-verbal reasoning skill. In the adaptation, the test plates (items) that are normally presented individually on a flipchart, were presented in sequence on a screen to whole classes, and children worked with a corresponding test booklet. Children were instructed to identify in their booklet the missing ‘piece’ of the main pattern array from the multiple distractors that were displayed on the screen. The plates were presented for the time durations described in the WRIT manual. The first 42 WRIT items were administered, as this set surpassed the typical discontinuation zone for the age groups in this study. The Cronbach’s alpha reliability was α = 0.85 for the monolingual sample and α = 0.78 for the bilingual sample. Given the adaptations for group administration, and because the WRIT battery is normed on a US population, grade-based normative scores were computed, based on the samples of children in the larger studies (*N* = 936 and *N* = 294). Standard scores were derived with a population mean of 100 and a standard deviation of 15.

#### Picture-Word Matching Test (PWM) – English Version (Caravolas et al., 2013, 2018a)

The PWM test measures children’s single word reading efficiency in a 3-min silent reading format; it was completed by all groups. The test contains 62 items that are cognates across several languages, and appear in frequency corpora of child-directed school texts across the languages (e.g., English: Masterson et al., 2003; French: Lété et al., 2004; Spanish: Martínez and García, 2004; Czech and Slovak: Kessler and Caravolas, 2011). The test items were presented in a booklet in order of increasing difficulty, and according to their appearance in school texts at specific school grades from Reception Year/Kindergarten to Year/Grade 6 (see Caravolas et al., 2013 for details). For each item, children viewed a target picture and then placed a tick under the word they selected as the best match to the picture from among four different printed words, which included the target item, a phonographemic distractor, a semantic distractor, and an unrelated distractor. Following a brief training session, children completed as many items as possible in 3 min. The score was the number of words selected correctly in 3 min. Test-retest reliability was not available for this test, however, in previous studies with younger English children (Caravolas et al., 2012, 2013) we found it to have high stability with *r*s ranging from 0.60 to 0.93. Year-group-based normative scores were computed, based on the samples of children in the larger studies (*N* = 936, and *N* = 294). Standard scores were derived with a population mean of 100 and a standard deviation of 15.

#### Picture-Word Matching Test (PWM)-Welsh Version (Caravolas et al., 2018b)

A Welsh version of the Picture-Word Matching test, parallel to the English version, was administered to the bilingual participants. The Welsh version was presented in the same order, according to the same method of administration as the English version. All children completed the Welsh version in a first testing session, and the English version in a subsequent session.

#### Test of English Single Word Spelling Ability

The Spelling subtest of the Wide Range Achievement Test IV (WRAT-IV; Wilkinson and Robertson, 2006) was adapted for group administration, adhering as closely as possible to the published guidelines, to the monolingual English participants only. It was not administered to the bilingual group due to constraints on the time available for testing in the bilingual schools. All monolingual participants spelled to dictation 13 alphabet letters followed by 36 words graded in difficulty and were given approximately 30 s to write each item. The cut-off of 36 words was selected as this corresponds to a standard score of 145 in grade six, and it was expected that most of sixth graders (equivalent to Year 5 in the United Kingdom) were unlikely to exceed this score. Each correct spelling was awarded one point and scoring was discontinued after 10 consecutive errors. The maximum possible score was 49. The Cronbach’s alpha reliability for this measure was α = 0.92. For the same reasons that applied to the Matrices test, grade-based normative scores were computed, based on the samples of North Walean children in the larger study of monolingual children (*N* = 936). Standard scores were derived with a population mean of 100 and a standard deviation of 15.

#### Test of Welsh Single Word Spelling Ability (Caravolas et al., 2018b)

This test was created to assess Welsh word spelling ability in primary-school-aged children. It contained 36 words, graded in terms of difficulty [per Welsh literacy curricula (Welsh Government, 2008)], and embedded in sentence contexts. Each word was repeated three times: once in isolation, the second time in a sentence context, and in isolation again, with approximately 30 s writing time allowed per word; all test items were administered. Participants were instructed to write each dictated word neatly in their booklets. Scoring was binary with one point for fully correct spellings and zero points otherwise. The Cronbach’s alpha reliability for this test was α = 0.82. Grade-based normative scores were computed based on the samples of children in the larger study (*N* = 294), and standard scores were derived with a population mean of 100 and a standard deviation of 15.

#### Spelling and Handwriting Legibility Test (SaHLT; Caravolas et al., in preparation)

Sentence spelling and handwriting legibility were measured using the SaHLT, a sentence dictation task that allows for the concurrent scoring of spelling and handwriting legibility.

##### Sentence Spelling

The ten sentences comprising the SaHLT are those used in Caravolas et al. (2005). For the purpose of this study, we used a shortened, eight-sentence (51 word), version of the test. The sentences varied in length from four to eight words, and within sentences, words varied from one to nine graphemes in length. Sentences increased in phonological, morphological, lexical, and orthographic complexity throughout the task. The item complexity increased in line with national spelling curriculum guidelines for England (cf. Caravolas et al., 2005). Spelling accuracy was measured in terms of binary conventional accuracy, with one point awarded for fully correct spellings, otherwise zero points; the possible maximum score was 51 points. A second, string edit distance, score was calculated for each spelling production using the computer software Ponto (Kessler, 2009). Ponto was set to apply a penalty of one point for each letter deletion, addition, and substitution within a word, thus generating a *letter distance* score (of the number of edits required to correct each spelling production). The number of penalties per word was averaged to derive a mean letter distance score per child.

##### Handwriting Legibility

Four separate dimensions of handwriting legibility were assessed per the guidelines in the SaHLT. The dimensions were developed based on a theoretical and empirical understanding of salient aspects of handwriting legibility (Caravolas et al., in preparation; Rosenblum et al., 2003). The dimensions are: (a) Letter Formation, which captures the child’s accuracy in producing the letter’s form, orientation, and consistency of its angle and size, (b) Letter Spacing, which measures the degree and consistency of the spacing between the letters within words, (c) Word Spacing, which – similarly to Letter Spacing – measures the degree and consistency of the spacing between words within a sentence, and (d) Line Alignment, which captures the degree and consistency with which the child writes the letters and words on the line. Each dimension is scored on a five-point Likert scale ranging from 1 (highly illegible) to 5 (highly legible), and an Overall Legibility score for each *sentence* is obtained by summing across the four-dimension scores, averaged over all sentences to a possible maximum of 20 points. On the abridged version of the SaHLT used in the present study, the five mean scores were generated by aggregating across the eight sentences. The SaHLT places no constraints on the type of script to be used; children are invited to write with pen or pencil and in print or cursive script, as is the norm in their schooling environment.

We have found this scale to be valid and reliable. For example, a strong correlation (*r* = 0.54) was observed between teacher responses on the Handwriting Proficiency Screening Questionnaire (Rosenblum, 2008) and SaHLT Overall Legibility, demonstrating convergent validity (Campbell and Fiske, 1959). The handwriting legibility measure showed excellent internal consistency (*α* = 0.97), test-retest (intraclass correlation = 0.76), and inter-rater (intraclass correlation = 0.83) reliabilities for the written productions of the monolingual English children. Children’s written productions were evaluated by trained scorers who had not scored the children’s spelling accuracy and were blind to their group classification. The bilingual children’s handwriting was evaluated by a different rater who received the same training as the rater of the monolingual sample. The inter-rater reliability was carried out by two trained scorers on a randomly selected sample of 25% of scripts from the bilingual group, and was found to be excellent (ICC = 0.85, *F*(31, 31) = 6.70, *p* < 0.001; Cicchetti, 1994).

### Procedure

Whole classes of children completed all the measures described above in specially prepared booklets in normal classroom conditions. For ease of administration and to reduce fatigue effects, measures were delivered over two 45- to 60-min sessions. The bilingual children completed one session through the medium of Welsh and the second through the medium of English, with at a minimum 1 hour elapsing between sessions. All sessions were conducted by a team of three or four trained research assistants who maintained good oversight of children’s work and of their compliance with the set instructions.

## Results

The data were analyzed in two main steps. First, between-group comparisons were conducted to investigate whether the bilingual children differed from their monolingual peers in literacy, and handwriting skills. Second, correlational and multi-group path analyses were conducted in order to assess the concurrent predictors of handwriting, and to test whether the predictive patterns were the same for all groups.

### Between-Group Comparisons

Preliminary data checking was carried out for each group on every measure. Outlier scores, representing 0.7% to 2% of the data, were Winsorized to within 2.7 SD of the respective group’s mean (Tukey, 1977). The resulting distributions were reasonably normal with the exception of the spelling measure of letter distance, which was positively skewed in all groups. Square root transformations normalized these distributions and the transformed scores were used in subsequent analyses. Descriptive statistics and analysis of variance results for group comparisons of age, non-verbal ability, and background literacy measures are reported in Table 2.

#### Group Matching Measures

Analyses of variance were carried out to test the anticipated effects on the variables used to match groups. By design, the bilingual (BIL) and chronological age-matched control (CA) groups did not differ in age, *t*(252) = 0.16, *p* < 0.435, *d* = 0.02, and both groups were significantly (on average 15 months) older than the spelling ability-matched control group (SA) (BIL vs. SA, *t*(252) = 11.37, *p* < 0.001, *d* = 1.43; CA vs. SA, *t*(252) = 11.21, *p* < 0.001, *d* = 1.41). Similarly, the analyses of spelling ability in sentence context (SaHLT), measured by binary accuracy, confirmed that the bilingual and spelling ability-matched groups did not differ from each other, *t*(252) = 0.08, *p* < 0.469, *d* = 0.01, and both groups spelled significantly less accurately than the CA controls (BIL vs. CA, *t*(252) = 4.25, *p* < 0.001, *d* = 0.53; CA vs. SA, *t*(252) = 4.18, *p* < 0.001, *d* = 0.52). Sentence spelling ability was also analyzed using the more refined measure of string edit letter distance to see whether the groups differed in the magnitude of their within-word error rates, which may have otherwise been missed by the binary scoring method. As reported in Table 2, this analysis replicated the pattern of results for the binary scoring method such that the CA group had the lower mean distance scores relative to bilingual, *t*(252) = 2.64, *p* = 0.004, *d* = 0.33, and SA groups, *t*(252) = 4.26, *p* < 0.001, *d* = 0.53, who in turn did not differ statistically from each other, *t*(252) = 1.85, *p* = 0.066, *d* = 0.23. No differences were found in standard scores on non-verbal ability between the bilingual and SA groups, *t*(248) = 1.96, *p* = 0.975, *d* = 0.25, or between the bilingual and CA groups, *t*(237) = 2.07, *p* = 0.980, *d* = 0.27, but the CA controls’ non-verbal ability was significantly lower than that of the SA controls, *t*(233) = 4.22, *p* < 0.001, *d* = 0.53, although, all groups performed within the average range. Consequently, in ensuing analyses we controlled for the potential moderating effect of non-verbal ability on literacy and handwriting attainments.

#### Background Literacy Measures

For a more complete picture of the literacy skills of the three groups, and to verify that all were performing within the normal range of their respective age and language of education contexts, additional reading and spelling measures were analyzed. ANCOVAs, controlling for potential effects of non-verbal abilities, were carried out on English word reading efficiency measured by the Picture-Word Matching test (PWM). Significant main effects were followed up with Bonferroni-corrected *post hoc* comparisons between groups. Because the same test was completed by all groups, we investigated the outcomes on both the raw scores (that is, the number of words matched correctly in 3 min), and the standardized score equivalents. The analysis of covariance (covarying non-verbal ability scores) on the raw scores revealed a main effect of the covariate *F*(1,357) = 25.88, *p* < 0.001, $\eta_{\rho }^{2}$ = 0.07, as well as a main effect of group such that, after controlling for the effect of non-verbal ability, the bilingual group read English words more efficiently than their monolingual peers (BIL vs. CA: *t*(251) = 4.78, *p* < 0.001, *d* = 0.68; BIL vs. SA: *t*(252) = 10.53, *p* < 0.001, *d* = 1.21) – a finding to which we return in the Discussion – and in turn, the younger SA group read less efficiently than their older monolingual peers (*t*(251) = −5.35, *p* < 0.001, *d* = 0.60). However, the ANCOVA on the standardized English Picture-Word Matching scores demonstrated that, after controlling for the significant effect of non-verbal ability (*F*(1,357) = 27.23, *p* < 0.001, $\eta_{\rho }^{2}$ = 0.07), the groups’ performances did not differ from each other, and furthermore, all groups were reading well within the normal range relative to their normative populations. The bilingual children had also completed the Welsh version of the Picture-Word Matching test, and thus we conducted an analysis comparing reading efficiency across groups when reading in their main language of instruction (English PWM for the CA and SA groups; Welsh PWM for the bilingual group). This was mainly done to ascertain that the bilingual group’s relatively strong performance on the English reading test did not reflect a group with particularly strong reading skills in their language of education. This ANCOVA on the standardized reading scores revealed that after controlling for a significant effect of non-verbal ability (*F*(1,356) = 27.51, *p* < 0.001, $\eta_{\rho }^{2}$ = 0.07), the three groups did not differ from each other, and all were reading well within the normal range.

In addition to assessments of reading, it was deemed important to assess spelling ability on an independent measure that was not used for participant selection and matching. All groups had been assessed on a graded single word spelling measure in their language of education; this was the WRAT Spelling Test in English and the Test of Welsh Single Word Spelling Ability. The raw results for these tests could not be compared directly, thus, we submitted the standardized score equivalents to an ANCOVA. As was true for the reading results, after controlling for the significant effect of non-verbal ability *F*(1,356) = 11.74, *p* < 0.001, $\eta_{\rho }^{2}$ = 0.03), the groups did not differ from each other in single word spelling ability, and all groups were performing well within the normal range relative to their normative population. These analyses confirmed that while in raw terms, the bilingual children spelled less well in English (SaHLT) than their monolingual counterparts, their spelling being on a par with monolingual children on average 15 months younger, all three groups represented typical readers and spellers in their educational and linguistic contexts.

#### Group Handwriting Legibility Profiles

To examine whether bilingual children’s handwriting differed from that of monolingual children, group performance was compared on each of the handwriting legibility dimensions. Owing to the high degree of relatedness among these dimensions (see correlations) we conducted a oneway MANCOVA with the four dimensions as dependent variables and group membership as the independent variable, covarying for non-verbal ability. The resulting model revealed performance on the handwriting legibility dimensions to differ significantly between groups, Pillai’s Trace = 0.20, *F*(8,712) = 9.83, *p* < 0.001.

Follow-up ANCOVAs on each dimension revealed significant group effects on letter formation, letter spacing, and line alignment, but not on word spacing (see Table 3 for the descriptive statistics and *post hoc* group comparisons). There were, however, different patterns of performance on each of the three dimensions, which were investigated using Bonferroni-corrected *post hoc* comparisons. On letter formation (after controlling for the significant covariate of non-verbal ability, *F*(1,358) = 7.51, *p* = 0.006, $\eta_{\rho }^{2}$ = 0.02), bilingual and spelling-ability-matched monolingual groups, who did not differ from each other (*t*(252) = 2.09, *p* = 0.113, *d* = 0.27), attained significantly lower scores than older monolingual controls (CA vs. BIL: *t*(252) = 5.27, *p* < 0.001, *d* = 0.66; CA vs. SA: *t*(252) = 2.09, *p* = 0.005, *d* = 0.47). On letter spacing, after controlling for non-verbal ability (*F*(1,358) = 8.19, *p* = 0.005, $\eta_{\rho }^{2}$ = 0.02), the bilingual group also received significantly lower scores than both monolingual control groups (CA vs. BIL: *t*(252) = 6.61, *p* < 0.001, *d* = 0.85; SA vs. BIL: *t*(252) = 4.28, *p* < 0.001, *d* = 0.51) who did not differ from each other (*t*(252) = 2.38, *p* = 0.054, *d* = 0.29). Similarly, for line alignment bilingual children received significantly lower scores than both monolingual control groups (CA vs. BIL: *t*(252) = 7.30, *p* < 0.001, *d* = 0.94; SA vs. BIL: *t*(252) = 4.24, *p* < 0.001, *d* = 0.51) and younger monolingual children received significantly lower scores than older monolinguals, (*t*(252) = 3.06, *p* = 0.007, *d* = 0.39). Finally, group comparisons on the overall legibility scores revealed that, after controlling for non-verbal ability, *F*(1,358) = 6.98, *p* = 0.009, *x*^2^ = 0.10, the bilingual group achieved a significantly lower overall score than the younger SA controls (*t*(252) = 2.97, *p* = 0.010, *d* = 0.37), and both groups had lower scores than their CA counterparts (CA vs. BIL: *t*(252) = 6.18, *p* < 0.001, *d* = 0.80; CA vs. SA: *t*(252) = 3.21, *p* = 0.004, *d* = 0.43; see Table 3). Overall, these analyses consistently showed the bilingual group to have poorer handwriting than monolingual children of their own age, and, in some cases, than younger children with a similar level of spelling ability.

**TABLE 3:** Summary of performance on handwriting legibility measures for bilinguals, chronological age-, and spelling ability-matched controls.

**Measure**	**CA**		**BIL**		**SA**		**Group comparisons**	
	***M***	***SD***	***M***	***SD***	***M***	***SD***	***F***	**$\eta_{\rho }^{2}$**
Letter formation	2.95	0.63	2.44	0.89	2.65	0.65	13.98***	0.07
Letter spacing	3.34	0.47	2.88	0.61	3.19	0.59	22.77***	0.11
Word spacing	3.40	0.53	3.29	0.65	3.27	0.64	1.60	0.01
Line alignment	3.75	0.55	3.20	0.64	3.52	0.64	26.85***	0.12
Overall legibility	13.45	1.72	11.80	2.35	12.63	2.05	19.13***	0.10

CA = chronological age-matched; BIL = bilinguals; SA = spelling ability-matched; ***p < 0.001.

### Relationships Between Spelling and Handwriting

#### Correlations

It is clear from the previous analyses that, despite having adequate spelling abilities for their age and education, the bilingual children had poorer handwriting legibility when writing in English. We investigated this further by examining the relationships between the background literacy skills and handwriting in monolingual and bilingual children. First, we examined the bivariate correlations between all variables of interest for each group separately; these are reported in Tables 4–6.

**TABLE 4:** Correlations between literacy and handwriting measures for chronological age-matched controls.

	**1**	**2**	**3**	**4**	**5**	**6**	**7**	**8**	**9**	**10**	**11**
(1) Age^*a*^											
(2) Non-verbal ability^*b*^	0.04										
(3) English word reading	0.48***	0.33***									
(4) L-ED word reading	0.48***	0.33***	1.00								
(5) L-ED word spelling^*b*^	0.03	0.25**	0.37***	0.37***							
(6) Sentence binary spelling	0.41***	0.26**	0.61***	0.61***	0.76***						
(7) Sentence letter distance	−0.45***	−0.27**	−0.63***	−0.63***	−0.72***	−0.96***					
(8) Letter formation	0.28***	0.15	0.25**	0.25**	0.30***	0.38*	−0.35***				
(9) Letter spacing	0.19*	0.20*	0.12	0.12	0.16	0.14	–0.15	0.65***			
(10) Word spacing	0.08	0.21*	0.15	0.15	0.14	0.15	–0.14	0.45***	0.43***		
(11) Line alignment	0.21*	0.12	0.26**	0.26**	0.13	0.20*	−0.23*	0.57***	0.41***	0.43***	
(12) Overall legibility	0.25**	0.21*	0.25**	0.25**	0.24**	0.29***	−0.28***	0.86***	0.78***	0.73***	0.77***

^*a*^Months; ^*b*^Standard scores. L-ED = language of education (i.e., English versions of PWM reading test and of the WRAT spelling test). ***p < 0.001, **p < 0.01, *p < 0.05.

**TABLE 5:** Correlations between literacy and handwriting measures for bilinguals.

	**1**	**2**	**3**	**4**	**5**	**6**	**7**	**8**	**9**	**10**	**11**
(1) Age^a^											
(2) Non-verbal ability^b^	–0.02										
(3) English word reading	0.37***	0.37***									
(4) L-ED word reading	0.33***	0.36***	0.80***								
(5) L-ED word spelling^b^	–0.01	0.23*	0.41***	0.44***							
(6) Sentence binary spelling	0.41***	0.33***	0.73***	0.67***	0.49***						
(7) Sentence letter distance	−0.39***	−0.37***	−0.72***	−0.69***	−0.54***	−0.94***					
(8) Letter formation	0.35***	0.16	0.43***	0.35***	0.39***	0.50***	−0.49***				
(9) Letter spacing	0.33***	0.11	0.32***	0.28**	0.28***	0.40***	−0.38***	0.74***			
(10) Word spacing	0.13	0.04	0.27**	0.15	0.17	0.21*	−0.19*	0.45***	0.58***		
(11) Line alignment	0.52***	0.11	0.37***	0.28**	0.18*	0.43***	−0.41***	0.71***	0.66***	0.51***	
(12) Overall legibility	0.40***	0.13	0.42***	0.33***	0.32***	0.47***	−0.45***	0.89***	0.88***	0.74***	0.85***

^*a*^Months; ^*b*^Standard scores. L-ED = language of education (i.e., Welsh versions of PWM reading test and of the Single Word Spelling test). ***p < 0.001, **p < 0.01, *p < 0.05.

**TABLE 6:** Correlations between literacy and handwriting measures for spelling ability-matched controls.

	**1**	**2**	**3**	**4**	**5**	**6**	**7**	**8**	**9**	**10**	**11**
(1) Age^a^											
(2) Non-verbal ability^b^	0.07										
(3) English word reading	0.64***	0.06									
(4) L-ED word reading	0.64***	0.06	1.00								
(5) L-ED word spelling^b^	0.20*	0.09	0.53***	0.53***							
(6) Sentence binary spelling	0.65***	0.17	0.80***	0.80***	0.73***						
(7) Sentence letter distance	−0.65***	–0.16	−0.79***	−0.79***	−0.74***	−0.97***					
(8) Letter formation	0.45***	0.10	0.50***	0.50***	0.40***	0.62***	−0.61***				
(9) Letter spacing	0.39***	0.17	0.33***	0.33***	0.24**	0.41***	−0.38***	0.57***			
(10) Word spacing	0.44***	0.04	0.44***	0.44***	0.35***	0.52***	−0.54***	0.61***	0.47***		
(11) Line alignment	0.36***	0.00	0.28***	0.28***	0.16	0.34***	−0.33***	0.61***	0.43***	0.56***	
(12) Overall legibility	0.51***	0.09	0.48***	0.48***	0.36***	0.58***	−0.58***	0.86***	0.75***	0.82***	0.81***

^*a*^Months; ^*b*^Standard scores. L-ED = language of education (i.e., English versions of PWM reading test and of the WRAT spelling test). ***p < 0.001, **p < 0.01, *p < 0.05.

Several noteworthy patterns emerged from these analyses. As expected, in all groups there were moderate to strong relationships between age and those literacy measures that had not been standardized, and hence already been controlled for age. Moderate correlations were also present between age and handwriting legibility measures. However, these correlations were stronger in the bilinguals and spelling-ability matched children, that is the relatively poorer spellers of English, than in older and better English speller (CA) group. Across all groups, non-verbal ability showed relatively weak associations with the handwriting legibility measures; in contrast, however, non-verbal skills associated moderately with the various literacy skills in the older (CA, BIL) but not the younger (SA) groups. As might be expected, all of the measures of reading and spelling intercorrelated relatively strongly in all the groups, however, literacy skills and handwriting dimension associations were weaker among the more advanced English spellers (CA group) than among the less advanced spellers of English (BIL and SA groups). Finally, all of the handwriting dimension scores intercorrelated relatively strongly within all groups. Looking at the individual dimensions, it is clear that letter formation had the most consistent relationships with literacy measures in all three groups. Again, these relationships were stronger in the poorer speller groups (BIL, SA) than among more advanced spellers (CA). In sum, with respect to the main question addressed in the present study, the patterns of correlations suggest that age (a proxy for amount of schooling and general writing experience) and literacy skills both seem to share variance with handwriting legibility; furthermore, their associations may be stronger among less advanced spellers (BIL, SA) than among more advanced spellers (CA).

#### Multigroup Path Models

We were interested in examining the extent to which spelling knowledge (task-specific and general), along with reading skills, non-verbal ability, and age, predicted handwriting legibility and whether these relationships differed in bilingual children, who were at once older but also poorer spellers of English, relative to their monolingual peers. Legibility was predicted in pairs of multigroup path models, the first always predicting *letter formation* because this measure was theoretically most likely to be related to spelling via letter knowledge (e.g., Longcamp et al., 2008), but also empirically it showed the most consistent correlations with spelling ability in the present study; the second model always predicted *overall legibility* as this captured the fuller handwriting legibility construct.

**FIGURE 1:**
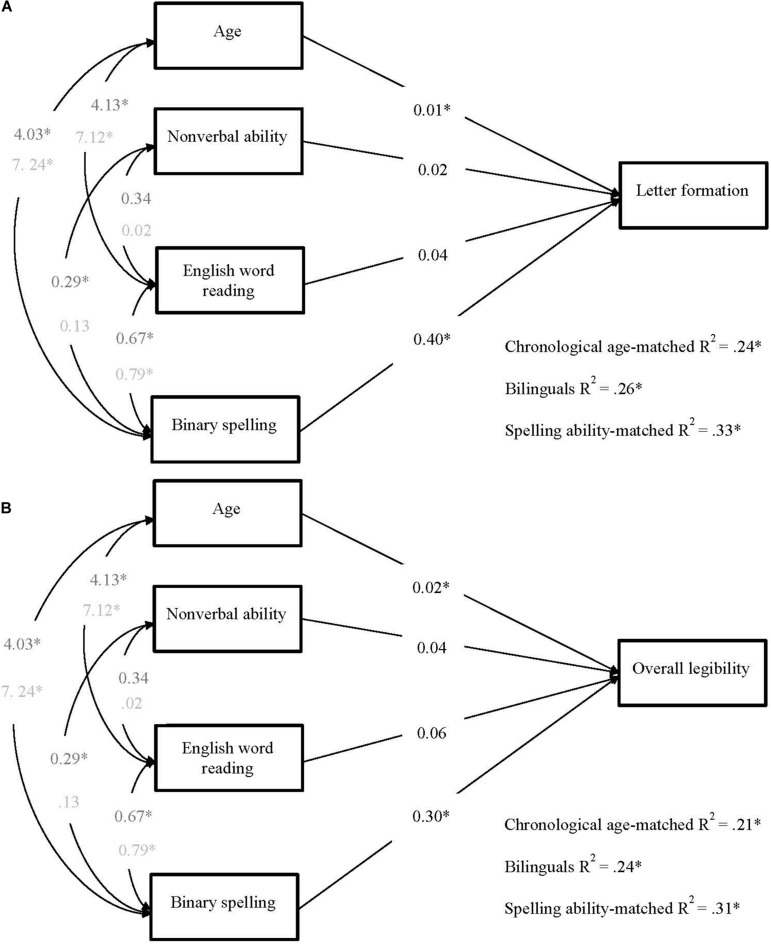
Multigroup path models predicting **(A)** letter formation and **(B)** total legibility in bilinguals, chronological age-, and spelling ability-matched controls. Each model included four predictors: age, non-verbal ability, English word reading, and binary sentence spelling. Unstandardized values are reported (*^∗^p* < 0.05). Covariance values in dark gray represent those for older, bilingual and chronological age-matched, children. Covariance values in light represent those for younger, spelling ability-matched children.

**FIGURE 2:**
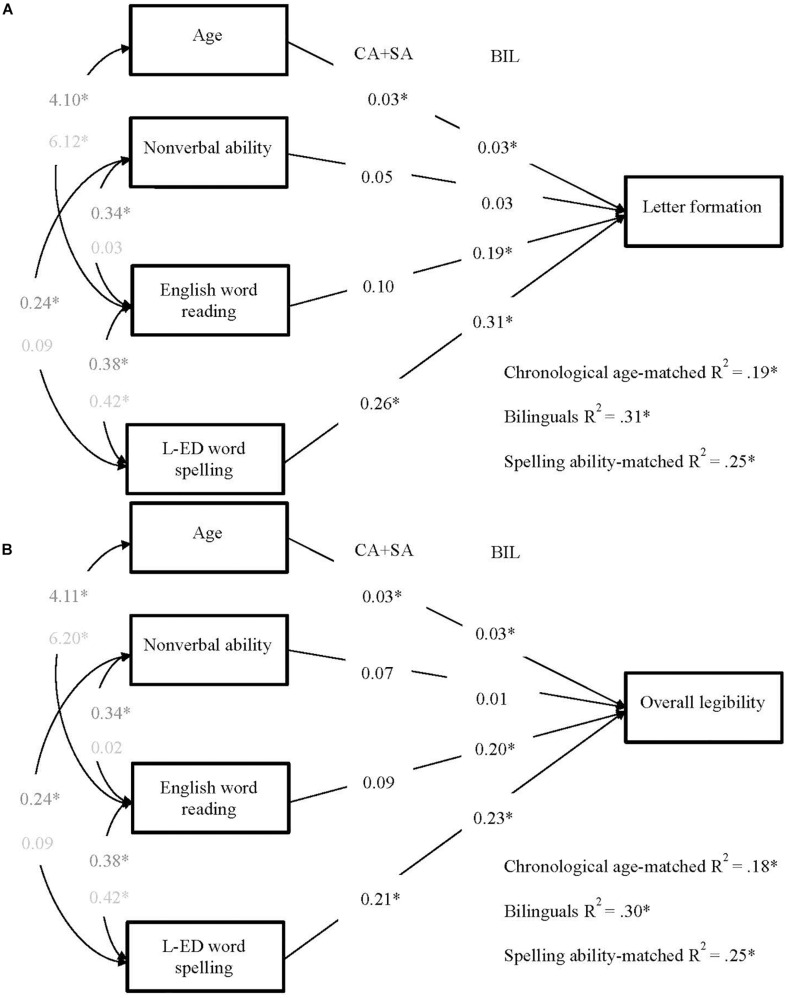
Multigroup path models predicting **(A)** letter formation and **(B)** total legibility in bilinguals, chronological age-, and spelling ability-matched controls. Each model included four predictors: age, non-verbal ability, English word reading, and L-ED word spelling (i.e., the WRAT spelling test for the monolingual groups, and the Welsh Single Word Spelling test for the bilingual group). Unstandardized values are reported (*^∗^p* < *0.05*). Path values on the left represent chronological age- and spelling ability-matched (CA + SA) weights and values on the right represent bilingual (BIL) weights. Covariance values in dark gray represent those for older, bilingual and chronological age-matched children. Covariance values in light represent those for younger, spelling ability-matched children. L-ED = language of education.

**FIGURE 3:**
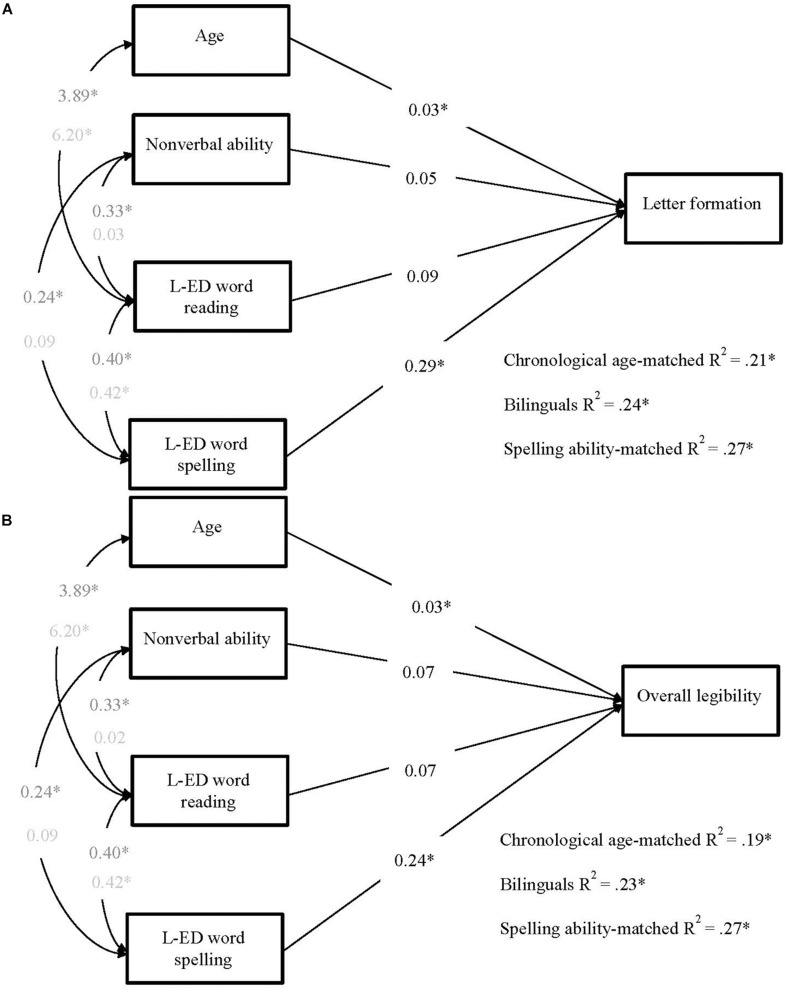
Multigroup path models predicting **(A)** letter formation and **(B)** total legibility in bilinguals, chronological age-, and spelling ability-matched controls. Each model included four predictors: age, non-verbal ability, L-ED word reading (i.e., English version of PWM reading test for the monolingual groups, and Welsh version of the PWM reading test for the bilingual group), and L-ED word spelling (i.e., English WRAT spelling test for the monolingual groups, and Welsh Single Word Spelling test for the Bilingual group). Unstandardized values are reported (*^∗^p* < 0.05). Covariance values in dark gray represent those for older, bilingual and chronological age-matched, children. Covariance values in light represent those for younger, spelling ability-matched children. L-ED = language of education.

In the first pair of models, the English spelling predictor reflected the binary accuracy score from the SaHLT, and the dependent measure was the letter formation legibility score (Figure 1A) and the overall handwriting legibility score (Figure 1B). Next, to investigate whether any predictive patterns between spelling and handwriting legibility would generalize beyond measures obtained from the SaHLT and the English language (in the case of the bilinguals), another pair of models was computed where spelling ability was measured by accuracy scores from the single word spelling task in each group’s language of education (English for the monolinguals and Welsh for the bilinguals), as reported in Figures 2A,B. These analyses were followed up by a pair of models with an additional manipulation on the predictor of reading, such that the English version of the PWM test was substituted by the Welsh version of this test (see Figures 3A,B).

Prior to the analyses, all variables were standardized within group. We conducted the multigroup path analyses in Mplus (Version 8.1; Muthén and Muthén, 2018). To deal with the small amount of missing data (< 5%), we used full-information maximum likelihood estimators. To test the multigroup goodness of fit, we used an iterative approach, where we first attempted to constrain unstandardized path weights to be equal across groups, followed by constraints on the covariances between predictors. Below, we report the final, best-fitting models, using this procedure.

##### Models in Which the SaHLT Spelling Measure Predicted SaHLT Handwriting Legibility

The models for the prediction of letter formation and total legibility are shown in Figure 1. Although non-verbal ability and English reading were not statistically significant predictors of either letter formation (Model 1A) or total legibility (Model 1B), they were kept in the model due to the significant covariances they shared with other predictors and because removing them was detrimental to the overall fit. Covariances between age and non-verbal ability were fixed at zero because non-verbal ability was standardized based on age. In the final models predicting letter formation, χ^2^ (16) = 9.13, *p* = 0.908, RMSEA = 0.000, SRMR = 0.052, CFI = 1.00, TLI = 1.05, and total legibility, χ^2^ (16) = 11.72, *p* = 0.762, RMSEA = 0.000, SRMR = 0.056, CFI = 1.00, TLI = 1.03, all path weights were constrained to be equal across all groups, and covariances were constrained to be equal across the groups of the older children (CAs and BIL) but not SAs.

The patterns of prediction were similar across models in that both age and binary spelling accuracy, but not non-verbal reasoning and reading, were significant unique predictors of handwriting. The strength of the predictors was similar across the bilingual and monolingual groups and spelling was the strongest predictor, especially so in the prediction of letter formation. The total variance explained in handwriting was similar and relatively small, but statistically significant in each of the groups.

##### Models in Which Single-Word Spelling Measures in Children’s Language of Education Predicted the SaHLT Handwriting Legibility

To investigate the role of children’s general spelling ability, as measured in their main language of education, we repeated the models described above but replaced the SaHLT spelling measure with single-word spelling measures in the group’s language of education (English for CA and SA groups, Welsh for the BIL group; see Figure 2). Both non-verbal ability and word spelling were standardized for age and so we fixed the covariances between age and these measures to zero. The final, best fitting, models of letter formation, χ^2^ (14) = 9.86, *p* = 0.772, RMSEA = 0.000, SRMR = 0.056, CFI = 1.00, TLI = 1.03, and total legibility, χ^2^ (14) = 10.91, *p* = 0.693, RMSEA = 0.000, SRMR = 0.058, CFI = 1.00, TLI = 1.03, were those in which path weights were constrained to be equal across monolingual children – but not bilinguals – and covariances were constrained to be equal across older children, but not SAs.

In these models, the patterns of predictions were the same for letter formation and overall legibility. Both age and single-word spelling accuracy were significant predictors of handwriting in all groups. While spelling remained the strongest predictor in all groups, its predictive strength was weaker than in the previous models. Interestingly, in the models of the bilingual group, when the measure of spelling ability was changed to Welsh single word spelling, English word reading emerged as an additional significant predictor of letter formation and overall legibility. The total variances explained in handwriting, significant in all cases, were similar in the monolingual groups and slightly elevated in the bilingual group, reflecting the additional predictor of English word reading. The comparison of predictive patterns across Models 1 and 2 suggested that the role of the spelling measures as predictors of handwriting legibility remained similar for all groups. Thus, when spelling was measured by the SaHLT in Model 1, its predictive strength was similar and strongest for all groups. When, in Model 2, spelling was measured by single word spelling tests (unrelated to the SaHLT), the predictive weight of spelling weakened for all groups, presumably reflecting the loss of some common method variance. To ascertain whether the English and Welsh single word spelling measures showed similar levels of association with the handwriting measures, we carried out follow-up Wald tests on the spelling path weights of the monolingual versus bilingual group. These confirmed no significant differences, in Model 2A (*W*(2) = 0.303, *p* = 0.859) and Model 2B (*W*(2) = 0.03, *p* = 0.859), suggesting that the differences in the language of the spelling tests did not bring about differences in the patterns of prediction for monolingual and bilingual children’s handwriting legibility. The variable that did increase in its predictive role from Model 1 to Model 2 was reading for the bilingual group.

The previous analysis revealed that, over and above word spelling in the children’s language of education, English word reading explained a significant amount of variance in handwriting in an English sentence dictation task among bilingual children but not monolingual children. The bilingual children’s model suggests that while general spelling knowledge – as measured by Welsh word spelling – continues to account for individual variations in handwriting on an English writing task, the writers are additionally drawing on English-specific orthographic knowledge, as reflected by the contributing effects from the English word reading measure. To test this hypothesis further, we repeated the same models, this time replacing in the bilingual group’s model, the English word reading efficiency with a parallel measure of Welsh word reading efficiency. We reasoned that if, in the former bilingual models, English reading was acting as a proxy for English orthographic knowledge, then replacing the reading measure for a Welsh one should lead to the loss of the effect of reading on handwriting.

The models predicting handwriting from age, non-verbal ability, word reading efficiency in the children’s language of education, and word spelling accuracy in the children’s language of education are shown in Figure 3. In the final models of letter formation, χ^2^ (18) = 11.83, *p* = 0.856, RMSEA = 0.000, SRMR = 0.070, CFI = 1.00, TLI = 1.04, and overall legibility, χ^2^ (18) = 12.79, *p* = 0.804, RMSEA = 0.000, SRMR = 0.072, CFI = 1.00, TLI = 1.04, path weights were constrained to be equal across all groups and covariances were constrained to be equal across older children, but not SAs.

Again, the same patterns of prediction held for letter formation as well as overall legibility. Like in the former models, age and word spelling were significant predictors of handwriting in all groups. However, replacing English word reading with Welsh word reading for the bilingual group led to the path between reading and handwriting to no longer be significant. Moreover, in the latter models, the relative weighting of spelling ability appeared to be weaker than in Models 1A and 1B, where spelling and handwriting measures are obtained from the same test. Similarly, the total amount of variance explained was somewhat lower than in the first two models, however, the fits between the respective Models 1 and 3 did not differ significantly (Models 1A vs. 3A χ^2^ = 2.70, Δ_*df*_ = 2, *p* = 0.259; models 1b vs. 3b, $\chi_{d\,i\,f\,f}^{2}$ = 1.07, Δ_*df*_ = 2, *p* = 0.586) suggesting that any differences are minimal.

## Discussion

In this study, we sought to better understand the influences of spelling ability and amount of handwriting experience and practice (estimated by years of schooling and age), on handwriting legibility. To do so, we compared typically developing children in Welsh-English bilingual education to peers in monolingual English education using an age- and spelling-ability-matched design. Thus, the spelling and handwriting skills of bilingual children in mid-primary school (class years 3–5) were compared to those of English monolingual groups of children of similar-age, years of schooling and non-verbal ability, on the one hand, and those some 15 months younger (class years 2–4), who were spelling at the same level as their bilingual peers.

Preliminary between-group comparisons of the children’s broader literacy skills, confirmed that on tests of silent word reading efficiency and of single word spelling – completed in each group’s main language of education (English for the monolingual groups and Welsh for the bilingual group) – all three groups were typical readers and spellers in their own age and educational contexts. One somewhat surprising finding was that the bilingual group read English words more efficiently (Picture-Word Matching test), in raw score terms, than their monolingual peers. This finding aligns with reports of facilitatory transfer effects in bilingual populations, especially those whose first or dominant written language is more consistent in terms of letter-sound mappings (e.g., Spanish, or in this case Welsh) than their second language (e.g., English; Durgunoğlu, 2002). For example, Spencer and Hanley (2003) found that Welsh-English bilingual children read English pseudo-words more accurately than their English monolingual age classmates, although the former group had less experience of reading English than the latter. However, we must interpret the present finding with caution because the bilingual group had completed the Welsh version of the Picture-Word Matching test in an earlier test session. Thus, they may have benefited from practice effects on the English test. While this reading result awaits further investigation, the important finding here is that, in relation to their normative populations, all groups read the English words well within the average range, and there were no significant differences between the groups in terms of mean standard scores.

On the critical measures of English spelling on the sentence dictation (SaHLT), as anticipated, the CA group spelled more accurately than the bilingual and SA groups, who in turn performed similarly to each other whether spelling ability was measured in terms of binary accuracy or Levenshtein letter edit distance. In sum, the bilingual group was well matched to the CA control group in terms of age and amount of schooling (i.e., presumably also on amount of handwriting experience), non-verbal ability and they had somewhat greater English word reading efficiency. Yet, their English spelling accuracy was significantly weaker, and on a par with 15-month younger English monolingual pupils.

Against this backdrop, we examined the mean ratings on the four handwriting legibility dimensions of the SaHLT (letter formation, letter spacing, word spacing, line alignment, as well as the aggregated overall legibility). With the exception of word spacing scores, which did not differentiate between groups, the bilingual group had consistently poorer legibility than their age mates, despite having otherwise comparable general and literacy skills. In comparison to their spelling ability peers, the bilingual group produced comparably legible letter forms, the dimension of handwriting that is likely most strongly related to spelling (Martlew, 1992; Longcamp et al., 2008; Caravolas et al., in preparation). However, on the remaining dimensions, they scored significantly less well, than their spelling-ability peers. This finding was contrary to our expectations that the dimensions of letter spacing and line alignment may be more indicative of handwriting components under motoric, and visuo-spatial control, and thereby be more amenable to the variations in handwriting experience and practice, reflected by age and number of years of schooling, be it in Welsh or in English. A possible explanation for the generally weak handwriting profile of the bilingual children is that handwriting legibility is to some extent dependent on orthography-specific practice and experience. That is, perhaps it is not experience with handwriting in general, but with the graphic/motor sequences of specific spelling patterns of words in an orthography.

We pursued this line of investigation in a series of multigroup path models. In the first model, we asked whether, over and above performance differences in the skills of interest, the predictors of handwriting vary as a function of spelling ability, age (a proxy for amount of handwriting experience), reading ability, and non-verbal reasoning. Importantly, we investigated whether the predictive patterns hold across age, ability, and language groups. In the first set of models, the measure of spelling was binary accuracy on the words of the SaHLT, and the measures of letter formation (Model 1A) and overall legibility (Model 1B) were also derived from the SaHLT. We found that spelling and to a lesser extent age predicted letter formation and overall legibility similarly across ability and language profiles, even when accounting for non-verbal reasoning and English reading abilities. This finding extends the well reported view that variations in spelling skills influence variations in handwriting fluency (e.g., Abbott and Berninger, 1993; Sumner et al., 2014; Afonso et al., 2018) and confirm that this relationship holds also for handwriting legibility. Turning to language profiles, these models explained performance in monolingual and bilingual children similarly which suggests that bilinguals use their English spelling skills in a similar way to their monolingual peers when handwriting in their second orthography. However, from this set of models, it remained unclear whether the effect of spelling was restricted to the SaHLT task, where the spelling and handwriting performance were derived from the same task. Also, this analysis was not informative about the generality of the spelling-handwriting relationship across different orthographies.

In the second set of models, we therefore replaced the binary sentence spelling score with a word spelling score of the child’s language of education (Welsh for bilinguals and English for monolinguals). These models (2A and 2B) revealed a slightly different picture. In both monolingual groups, single word spelling and – to a lesser extent – age were the only significant predictors of letter formation and overall legibility, thus replicating the first set of models. However, among the bilingual group, English word reading abilities emerged as a predictor of letter formation and overall legibility, in addition to age and Welsh word spelling. The prediction of handwriting from a separate spelling measure further strengthens the view that the influence of spelling ability on handwriting legibility generalizes beyond specific tasks – although the influence of spelling in the first analysis tended to be stronger than in the second, suggesting that common method variance accounted for additional variance when both spelling and handwriting were measured by the same test. Furthermore, these models suggest that bilingual writers’ handwriting is influenced by some general spelling ability, as demonstrated by the significant path between Welsh spelling knowledge and legibility. In addition, the significant path between English reading and legibility – which was only significant in the bilingual group – suggests that bilinguals were utilizing some English orthography-specific knowledge to shape the legibility of their English handwriting.

In the final set of models (3A and 3B), we further tested the hypothesis that bilingual children were drawing on some orthography-specific knowledge when writing in their second orthography by replacing the English word reading measure with its Welsh analog, thus removing any measure of English orthography knowledge in the bilingual group’s model. This manipulation led to the loss of the significant path between word reading and handwriting, present for the bilingual group in models 2A and 2B. This finding strengthens our interpretation of Models 2A and 2B and suggests that when Welsh-English bilingual children handwrite in English, they rely on general spelling knowledge as well as orthography-specific spelling, and orthographic knowledge. This interpretation, in line with current theorizing about the organization of the bilingual lexicon (e.g., Kroll et al., 2005; de Groot, 2011), implies that during handwritten spelling production, bilingual writers may rely on orthographic (spelling) knowledge specific to the language in use (i.e., English or Welsh), in addition to relying on a language-general or integrated construct of “general orthographic knowledge” and both of these sources of knowledge may then have a downstream effect on the quality of handwriting legibility. Thus in our Model 2, the effect of the language-general/integrated knowledge may be estimated by the path from spelling to handwriting legibility for the bilingual group, whereas any residual orthography-specific knowledge may be estimated by the path from reading to handwriting legibility in Models 2 and 3 for all groups. For the monolingual groups, language-specific spelling knowledge completely overlaps with our putative “language-general or integrated spelling/orthographic knowledge” construct, and for this reason English reading ability does not contribute to handwriting over and above English spelling ability.

In sum, the present study of group and individual differences indicates that spelling ability, more so than variables related to the amount of practice in handwriting, such as age and hence the amount of schooling experience, exerts a relatively strong and stable influence on handwriting skills, including legibility. However, variations in age did additionally make consistent contributions to handwriting legibility skills. These results were obtained in comparisons of more and less advanced spellers, all of whom were typically developing. The finding that the bilingual group had weaker spelling skills and handwriting skills in English, on a par with 15-month-younger monolingual children suggests that the contingency between spelling ability and handwriting is to a large extent driven by the amount of experience and practice in writing a *specific* orthography, and not only by spelling disorder. It could be argued that despite their otherwise adequate reading and spelling skills in their main language of education (Welsh), the bilingual children were simply worse at handwriting than their monolingual counterparts. To follow up this possibility, we used the method and criteria of the SaHLT for all but the word spacing dimension, to evaluate the handwriting legibility of 57 randomly selected participants on the 36 words of Welsh Single Word Spelling test, and we compared these to their scores on the English SaHLT. The analysis showed superior handwriting scores on the Welsh test for every dimension. Certainly, this last finding requires replication with fully analogous measures, including a Welsh version of the SaHLT as well as an English single word spelling test; but, this initial analysis is suggestive of the orthography-specific writing experience hypothesis. Finally, it is important to note that the total amount of variance was significant and consistent across all models but was relatively small in size (*R*^2^ = 0.19–0.33). We expect that the inclusion of measures of other skills believed to affect handwriting ability, such as motor- and attention-related skills (e.g., Adi-Japha et al., 2007; Prunty and Barnett, 2019), as well as more direct measures of the amount of handwriting experience of the participants would substantially increase the amount of variance explained. These extensions to the present work await further research.

Our study has some implications for educational practice. The legibility of children’s writing impacts their educational experiences and outcomes (e.g., Feder and Majnemer, 2007), and thus it is important for educators to understand the causes and possible steps to remediating poor legibility. The present study shows clearly that handwriting legibility improves with spelling ability more so than with the handwriting practice that accrues with years of schooling and maturation. Moreover, our study suggests that it is learning to write the specific orthographic patterns of a given language that is particularly beneficial to handwriting development. Thus, it seems advisable for educators to focus on handwriting legibility, not only in dedicated handwriting lessons, but also during spelling instruction, and for bilingually educated children, and second language/orthography learners, handwriting should be a focus during spelling instruction in both taught languages. In addition, during dedicated handwriting practice, it would be beneficial to include spelling patterns of the language(s) of education. That is, taking the Welsh-English example, while handwriting skills acquired in the context of Welsh literacy lessons should generalize to some extent to handwriting quality in English, our results suggest that English spelling practice may confer even stronger benefits on handwriting in English. Finally, when children present with poor handwriting, this may be a signal to teachers of underlying spelling difficulties.

## Data Availability Statement

The datasets for this article are not publicly available because the data set is new, part of a larger data set, and still being exploited by the authors. The data will be made available in the future. Requests to access the datasets should be directed to MC, m.caravolas@bangor.ac.uk.

## Ethics Statement

The studies underpinning this article were reviewed and approved by Ethics Committee of the School of Psychology, Bangor University, United Kingdom. Written informed consent was provided by headteachers. Participants’ legal guardian/next-of-kin gave opt-out consent in accordance with the national legislation and the institutional requirements.

## Author Contributions

MC conceptualized the aims, design of the study, and led the write-up of the manuscript. CD contributed to all aspects of the manuscript development and was the main contributor to the data analysis. CH, CW, and CD performed data collection and scoring. CH and CW contributed to specific sections of the manuscript. All authors read and approved the submitted version.

## Conflict of Interest

The authors declare that the research was conducted in the absence of any commercial or financial relationships that could be construed as a potential conflict of interest.

## References

[B1] Abbott R. D. Berninger V. W. (1993). Structural equation modeling of relationships among developmental skills and writing skills in primary- and intermediate-grade writers. *J. Educ. Psychol.* 85 478–508. 10.1037//0022-0663.85.3.478

[B2] Adi-Japha E. Landau Y. E. Frenkel L. Teicher M. Gross-tsur V. Shalev R. S. (2007). ADHD and dysgraphia: underlying mechanisms. *Cortex* 43 700–709. 10.1016/S0010-9452(08)70499-4 17710822

[B3] Afonso O. Suárez-Coalla P. Cuetos F. (2019). Writing impairments in Spanish children with developmental dyslexia. *J. Learn. Disabil.* 53 109–119. 10.1177/0022219419876255 31526093

[B4] Afonso O. Suárez-Coalla P. González-Martín A. Cuetos F. (2018). The impact of word frequency on peripheral processes during handwriting: a matter of age. *Q. J. Exp. Psychol.* 71 695–703. 10.1080/17470218.2016.1275713 28054498

[B5] Alamargot D. Morin M. F. (2015). Does handwriting on a tablet screen affect students’ graphomotor execution? A comparison between grades two and nine. *Hum. Mov. Sci.* 44 32–41. 10.1016/j.humov.2015.08.011 26298215

[B6] Arfé B. Corato F. Pizzocaro E. Merella A. (2019). The effects of script and orthographic complexity on the handwriting and spelling performance of children with Dyslexia. *J. Learn. Disabil.* 53 96–108. 10.1177/0022219419892845 31823679

[B7] Berninger V. Winn W. (2006). “Implications of advancements in brain research and technology for writing development, writing instruction, and educational evolution,” in *Handbook of Writing Research*, eds MacArthur C. A. Graham S. Fitzgerald J. (New York, NY: Guilford Publications), 96–114.

[B8] Berninger V. Yates C. Cartwright A. Rutberg J. Remy E. Abbott R. (1992). Lower-level developmental skills in beginning writing. *Read. Writ.* 4 257–280.

[B9] Berninger V. W. Amtmann D. (2003). “Preventing written expression disabilities through early and continuing assessment and intervention for handwriting and/or spelling problems: research into practice,” in *The Handbook of Learning Disabilities*, eds Swanson H. L. Harris K. Graham S. (New York, NY: Guilford Publications), 345–363.

[B10] Berninger V. W. Swanson H. L. (1994). “Modifying Hayes and Flower’s model of skilled writing to explain beginning and developing writing,” in *Advances in Cognition and Educational Practice*, Vol. 2 eds Butterfield E. C. Carlson J. S. (Stamford, CT: JAI Press), 57–81.

[B11] Campbell D. T. Fiske D. W. (1959). Convergent and discriminant validation by the multitrait-multimethod matrix. *Psychol. Bull.* 56 81–105. 13634291

[B12] Caravolas M. Lervåg A. Defior S. Seidlová Málková G. Hulme C. (2013). Different patterns, but equivalent predictors, of growth in reading in consistent and inconsistent orthographies. *Psychol. Sci.* 24 1398–1407. 10.1177/0956797612473122 23744876

[B13] Caravolas M. Lervåg A. Mousikou P. Efrim C. Litavsky M. Onochie-Quintanilla E. (2012). Common patterns of prediction of literacy development in different alphabetic orthographies. *Psychol. Sci.* 23 678–686. 10.1177/0956797611434536 22555967 PMC3724272

[B100] Caravolas M. Mikulajová M. Defior S. Seidlová Málková G. (2018a). *Multilanguage Assessment Battery of Early Literacy.* Available online at: https://www.eldel-mabel.net/test/

[B101] Caravolas M. Mikulajová M. Defior S. Seidlová Málková G. (2018b). *Multilanguage Assessment Battery of Early Literacy.* Available online at: https://www.eldel-mabel.net/cy/test/

[B15] Caravolas M. Volín J. Hulme C. (2005). Phoneme awareness is a key component of alphabetic literacy skills in consistent and inconsistent orthographies: evidence from Czech and English children. *J. Exp. Child Psychol.* 92 107–139. 10.1016/j.jecp.2005.04.003 16038927

[B16] Cicchetti D. V. (1994). Guidelines, criteria, and rules of thumb for evaluating normed and standardized assessment instruments in psychology. *Psychol. Assess.* 6 284–290.

[B17] Connelly V. Campbell S. Maclean M. Barnes J. (2006). Contribution of lower order skills to the written composition of college students with and without dyslexia. *Dev. Neuropsychol.* 29 37–41. 10.1207/s15326942dn2901 16390293

[B18] Cooke A. (2002). Case study: a virtual non-reader achieves a degree. *Dyslexia* 8 102–115. 10.1002/dys.217 12067184

[B19] Delattre M. Bonin P. Barry C. (2006). Written spelling to dictation: sound-to-spelling regularity affects both writing latencies and durations. *J. Exp. Psychol. Learn. Mem. Cogn.* 32, 1330–1340. 10.1037/0278-7393.32.6.1330 17087587

[B20] de Groot A. M. (2011). *Language and Cognition in Bilinguals and Multilinguals: An Introduction.* New York, NY: Psychology Press.

[B21] Downing C. Caravolas M. (2018). “Handwriting legibility reflects spelling difficulties in dyslexia but not in developmental coordination disorder (DCD),” in *Poster Presented at the Meeting of the Society for the Scientific Study of Reading’s 25th Annual Meeting*, Brighton.

[B22] Durgunoğlu A. Y. (2002). Cross-linguistic transfer in literacy development and implications for language learners. *Ann. Dyslexia* 52 189–204. 17625051

[B23] Feder K. P. Majnemer A. (2007). Handwriting development, competency, and intervention. *Dev. Med. Child Neurol.* 49 312–317. 17376144 10.1111/j.1469-8749.2007.00312.x

[B24] Glutting J. J. Adams W. Sheslow D. (2000). *Wide Range Intelligence Test [Assessment instrument].* Wilmington, DE: Wide Range.

[B25] Graham S. Berninger V. W. Abbott R. D. Abbott S. P. Whitaker D. (1997). Role of mechanics in composing of elementary school students: a new methodological approach. *J. Educ. Psychol.* 89 170–182. 10.1037/0022-0663.89.1.170

[B26] Jones D. Christensen C. A. (1999). Relationship between automaticity in handwriting and students’ ability to generate written text. *J. Educ. Psychol.* 91 44–49.

[B27] Juel C. (1988). Learning to read and write: a longitudinal study of 54 children from first through fourth grades. *J. Educ. Psychol.* 80 437–447. 10.1037/0022-0663.80.4.437

[B28] Juel C. Griffith P. L. Gough P. B. (1986). Acquisition of literacy: a longitudinal study of children in first and second grade. *J. Educ. Psychol.* 78 243–255. 10.1016/j.ridd.2013.02.001 23500170

[B29] Kandel S. Lassus-sangosse D. Grosjacques G. Perret C. (2017). The impact of developmental dyslexia and dysgraphia on movement production during word writing. *Cogn. Neuropsychol.* 34 219–251. 10.1080/02643294.2017.1389706 29157129

[B30] Kandel S. Perret C. (2015). How does the interaction between spelling and motor processes build up during writing acquisition? *Cognition* 136 325–336. 10.1016/j.cognition.2014.11.014 25525970

[B31] Kessler B. (2009). *Ponto [ComputerSoftware].* Available online at: http://spell.psychology.wustl.edu/ponto (accessed August, 2019).

[B32] Kessler B. Caravolas M. (2011). *Weslalex: West Slavic Lexicon of Child-Directed Printed Words.* Available online at: http://spell.psychology.wustl.edu/weslalex (accessed August, 2019).

[B33] Kroll J. F. Sumutka B. M. Schwartz A. I. (2005). A cognitive view of the bilingual lexicon: reading and speaking words in two languages. *Int. J. Biling.* 9, 27–48. 10.1177/13670069050090010301

[B34] Lambert E. Quémart P. (2019). Introduction to the special issue on the dynamics of written word production: methods, models, and processing units. *Read. Writ.* 32 1–12.

[B35] Lété B. Sprenger-Charolles L. Colé P. (2004). MANULEX: a grade-level lexical database from French elementary school readers. *Behav. Res. Methods Instr. Comput.* 36 156–166. 15190710 10.3758/bf03195560

[B36] Longcamp M. Boucard C. Gilhodes J. C. Anton J. L. Roth M. Nazarian B. (2008). Learning through hand-or typewriting influences visual recognition of new graphic shapes: behavioral and functional imaging evidence. *J. Cogn. Neurosci.* 20 802–815. 10.1162/jocn.2008.20504 18201124

[B37] Martínez J. A. García M. E. (2004). *Diccionario de Frecuencias del Castellano Escrito en Niños de 6 a 12 años [Dictionary of frequencies of printed Spanish words for children aged 6 to 12].* Salamanca: Servicio de Publicaciones Universidad Pontificia de Salamanca.

[B38] Martlew M. (1992). Handwriting and spelling: Dyslexic children’s abilities compared with children of the same chronological age and younger children of the same spelling level. *Br. J. Educ. Psychol.* 625 375–390. 10.1111/j.2044-8279.1992.tb01030.x 1467257

[B39] Masterson J. Stuart M. Dixon M. Lovejoy S. (2003). *Children’s Printed Word Database.* Available online at: http://www.essex.ac.uk/psychology/cpwd (accessed August, 2019). 10.1348/000712608X371744 20021708

[B40] Miller L. T. Missiuna C. A. Macnab J. J. Malloy-Miller T. Polatajko H. J. (2001). Clinical description of children with developmental coordination disorder. *Can. J. Occup. Ther.* 68 5–15. 10.1177/000841740106800101 11233688

[B41] Muthén L. K. Muthén B. O. (2018). *Mplus User’s Guide*, 8th Edn Los Angeles, CA: Muthén & Muthén.

[B42] Olive T. (2014). Toward a parallel and cascading model of the writing system: a review of research on writing processes coordination. *J. Writ. Res.* 6 173–194.

[B43] Pontart V. Bidet-Ildei C. Lambert E. Morisset P. Flouret L. Alamargot D. (2013). Influence of handwriting skills during spelling in primary and lower secondary grades. *Front. Psychol.* 4:818. 10.3389/fpsyg.2013.00818 24204357 PMC3817363

[B44] Prunty M. Barnett A. L. (2019). Accuracy and consistency of letter formation in children with developmental coordination disorder. *J. Learn. Disabil.* 53 120–130. 10.1177/0022219419892851 31833795 PMC7008555

[B45] Puranik C. S. Al Otaiba S. (2012). Examining the contribution of handwriting and spelling to written expression in kindergarten children. *Read. Writ.* 25 1523–1546. 10.1007/s11145-011-9331-x 23087544 PMC3474373

[B46] Rosenblum S. (2008). Development, reliability, and validity of the Handwriting Proficiency Screening Questionnaire (HPSQ). *Am. J. Occup. Ther.* 62 298–307. 18557006 10.5014/ajot.62.3.298

[B47] Rosenblum S. Weiss P. L. Parush S. (2003). Product and process evaluation of handwriting difficulties: a review. *Educ. Psychol. Rev.* 15 41–81.

[B48] Roux S. McKeeff T. J. Grosjacques G. Afonso O. Kandel S. (2013). The interaction between central and peripheral processes in handwriting production. *Cognition* 127 235–241. 10.1016/j.cognition.2012.12.009 23454797

[B49] Søvik N. Samuelstuen M. Svarva K. Lie A. (1996). The relationship between linguistic characteristics and reading/writing performances of Norwegian children. *Read. Writ.* 8 199–216.

[B50] Spencer L. H. Hanley J. R. (2003). Effects of orthographic transparency on reading and phoneme awareness in children learning to read in Wales. *Br. J. Psychol.* 94 1–28. 10.1348/000712603762842075 12648386

[B51] Stanovich K. E. (1986). Matthew effects in reading: some consequences of individual differences in the acquisition of literacy. *Read. Res. Q.* 21 360–406.

[B52] Sumner E. Connelly V. Barnett A. L. (2012). Children with dyslexia are slow writers because they pause more often and not because they are slow at handwriting execution. *Read. Writ.* 26 991–1008. 10.1007/s11145-012-9403-6

[B53] Sumner E. Connelly V. Barnett A. L. (2014). The influence of spelling ability on handwriting production: children with and without dyslexia. *J. Exp. Psychol.* 40 1441–1447. 10.1037/a0035785 24548322

[B54] Tukey J. W. (1977). *Exploratory Data Analysis*, Vol. 2 Reading: Addison-Wesley.

[B55] van Galen G. P. (1991). Handwriting: psychomotor issues for a theory. *Hum. Mov. Sci.* 10 165–191. 10.1016/j.cognition.2016.01.011 26803393

[B56] Welsh Government (2007). *Defining Schools According to Welsh Medium Provision.* Cardiff: Welsh Government.

[B57] Welsh Government (2008). *Curriculum for Wales 2008.* Cardiff: Welsh Government.

[B58] Wilkinson G. S. Robertson G. J. (2006). *The Wide Range Achievement Test–4 (WRAT-4) [Assessment instrument]*. Lutz, FL: Psychological Assessment Resources.

